# Standards of Beauty

**Published:** 1891-08

**Authors:** 


### STANDARDS OF BEAUTY.

Standards of beauty are different in different ages. Fashion alone not only changes ideas of graceful form but she changes the human form itself. A physiologist shows that since the introduction of the corset, "the perfect feminine figure" in art has changed. Even the nude woman of the nineteenth century is different from the model of past centuries. The Venus of Milo, which since her discovery in the Island of Milos, in 1820, has been accepted as the most perfect ancient type of beauty, would measure about as follows, figured down to a woman of 5 feet 4 inches high: Neck, 14⅞ inches; bust, 36½ inches; immediately under bust, 31 inches; so-called waist line, 30 inches; hips, 42 inches; length of foot, 9⅝ inches; upper arm, 12½ inches. The strong point of comparison with modern models is that there is a difference of but six and a half inches between the waist measure and bust measure. The sculptor of the Venus believed in a full waist and a small bust. His goddess, should she shrink to five feet four inches in height, would wear a No. 6 shoe. Most of the great painters of the earlier days agree with the sculptors. The nude figure in Titian's "Earthly and Heavenly Love" has an extremely slight curve at the waist line, and there are dozens of illustrations to the same ideal.

